# Changes in the cochlear and retrocochlear parts of the auditory system in 19–39 and 40–60 years old patients with type 1 diabetes mellitus

**DOI:** 10.1371/journal.pone.0285740

**Published:** 2023-05-19

**Authors:** Mihály Horváth, Zoltán Herold, Marianna Küstel, László Tamás, Péter Prekopp, Anikó Somogyi, Anita Gáborján

**Affiliations:** 1 Department of Otorhinolaryngology, Head- and Neck Surgery, Semmelweis University, Budapest, Hungary; 2 Department of Internal Medicine and Oncology, Division of Oncology, Semmelweis University, Budapest, Hungary; 3 Department of Voice, Speech and Swallowing Therapy, Faculty of Health Sciences, Semmelweis University, Budapest, Hungary; 4 Department of Internal Medicine and Hematology, Semmelweis University, Budapest, Hungary; All India Institute of Speech and Hearing, INDIA

## Abstract

Pathophysiological alterations in the cochlea and functional tests of the auditory pathway support that in diabetes both vasculopathy and neural changes could be present. The aim of our research was to study the differential effect of type 1 diabetes mellitus (T1DM) on two different age groups. Audiological investigation was carried out in 42 patients and 25 controls at the same age groups. Investigation of the conductive and sensorineural part of the hearing system by pure tone audiometry, distortion product otoacoustic emission measurement and acoustically evoked brainstem response registration were evaluated. Among the 19-39-year-old people the incidence of hearing impairment was not different in the diabetes and control groups. Among the 40-60-year-old people hearing impairment was more common in the diabetes group (75%) than in the control group (15,4%). Among patients with type 1 diabetes, the mean threshold values were higher in both age groups at all frequencies although significant difference was in 19–39 years old group: 500-4000Hz right ear, 4000Hz left ear, in 40–60 years old group: 4000–8000 Hz both ears. In the 19–39 years old diabetes group only at 8000 Hertz on the left side was a significant (p<0,05) difference in otoacoustic emissions. In the 40–60 years old diabetes group significantly less otoacoustic emissions at 8000 Hz on the right side (p<0,01) and at 4000-6000-8000 Hertz on the left side, (p<0,05, p<0,01, p<0,05 respectively) was present compared to the control group. According to ABR (auditory brainstem response) latencies and wave morphologies, a possible retrocochlear lesion arose in 15% of the 19–39 years old and 25% of the 40–60 years old diabetes group. According to our results, T1DM affects negatively the cochlear function and the neural part of the hearing system. The alterations are more and more detectable with aging.

## Introduction

As one of our most important senses, the ability to hear enables us to connect to the world for many very important, even vital, reasons. Most importantly, hearing connects us to people enabling us to communicate in a way that none of our other senses can achieve. As the famed 20th-century activist and educator, Helen Keller, once said, “Blindness cuts us off from things, but deafness cuts us off from people”. People who can’t hear well may become depressed, or they may withdraw from others because they feel frustrated or embarrassed about not understanding what is being said. Around the world, approximately more than 1,5 billion people have impaired hearing [1]. Hearing loss can be classified according to severity into the following categories: slight 16–25 dB, mild 26–40 dB, moderate 41–55 dB, moderately severe 56–70 dB, severe 71–90 dB and profound above 91 dB [2].

The alterations in the hearing system can be detected at a very young age. In a recent study, the hearing of children with Type 1 diabetes and healthy participants were evaluated. The hearing thresholds in both groups were within the normal range. The mean hearing thresholds (500-1000-2000-4000 Hz) were higher, the distortion product of otoacoustic emission (OAE) amplitudes were lower, and the interpeak latencies of I—V and III -V waves were delayed in the diabetic group [3]. According to the literature, there is a connection between hearing loss and social isolation, and loneliness in both the elderly and the young [4, 5]. Regular hearing screening and proper hearing aid supply—or cochlear implantation—can prevent or improve the above problems.

Diabetes mellitus is a chronic, metabolic disorder, which is characterized by elevated blood glucose level due to the impairment or the complete loss of insulin, which over time leads to serious damage of several tissues and organs. Diabetes is one of the most prevalent diseases in our time, affecting approximately 8–9% of the world’s population. Its current classification includes two major forms: type 1 diabetes mellitus (T1DM) and type 2 diabetes mellitus (T2DM) [6]. T1DM accounts for 5–10% of all diabetes patients. In the T1DM patients, β-cells of the pancreas are gradually destroyed by an autoimmune process. The autoimmune form shows a strong HLA (human leukocyte antigen) association. Some genes have a protective effect, while others pose an increased risk of developing the disease. The number of insulin-producing pancreatic cells decreases relatively rapidly and several antibodies play a role in this mechanism. Of these, the most common are insulin autoantibody, glutamic-acid-decarboxylase antibody anti-GAD, anti-tyrosine phosphatase islet cell antibody and zinc transporter 8 antibody. T1DM accounts for the majority of young-onset diabetes cases. It is important, however, to highlight that T1DM may also come to light in later ages as well, that form of T1DM is called latent autoimmune diabetes in adults (LADA) [6].

Hearing trouble occurs in 40 percent of the population over 65 years of age. 60 percent of the elderly do not understand normal speech due to hearing loss [7]. Damage to similar structures has been observed during the pathological processing of the cochlea of type 1 diabetic patients and individuals showing signs of presbycusis. In both cases lesions in the stria vascularis and loss of outer hair cells can be seen. Among elderly healthy participants, a smaller number of spiral ganglion cells, while in the case of T1DM, the thickening of the wall of the cochlear spiral modiolar artery that supplies them was observed [8–10]. Clinical studies have focused more on the role of central structures and neuropathy during the examination of the brainstem waves [11–13]. These studies suggest that both microvascular causes and neuropathy–which are common complications in T1DM–are likely to play a role in the detrimental effects of T1DM on the auditory system, although the exact pathomechanism is not fully known, more and more details become clearer.

The central auditory nervous system consists of many neural structures from the vestibulocochlear nerve to the auditory cortex. When the stimulus travels through these structures each of them generates electrical signals. These responses can be detected with electrodes attached to the patient’s head and visualized as waves. These waves can be analyzed with a computer. The amplitude of the waves and absolute and interpeak -for example I-III, I-V- latencies are also informative. Interpeak latencies are the time between waves. It refers to the conduction of neural pathways. The information of the wave producing structure is mainly based on measurements in animals. Later, during intraoperative tests, previous information was refined. According to this, each wave is generated by the following neural structures: (Table 1) [14–17].

**Table 1:** Brainstem-evoked response waves, and their origin.

WAVE	I-II	III	IV	V	VI-VII
**STRUCTURE**	vestibulocochlear nerve	nucleus cohlearis	oliva superior	lemniscus lateralis	colliculus inferior

More and more evidence supports the theory, that retrocochlear neural structures can be damaged in type 1 diabetes. In a group of T1DM patients, the pure tone audiometry thresholds were higher than in the control group. As proof of normal cochlear function, there wasn’t a significant difference in otoacoustic emissions compared to controls and in the ABR measurement, the first wave was also in the normal range. The III and V waves—which are generated in the brainstem- were significantly delayed. These findings aid the hypothesis of neural changes in diabetes [18]. In one of the early researches where ABR was used, they examined middle-aged T1DM patients by finding the latency of waves I and II to be within the normal range, while the time interval between waves I-III and I-V was found to be delayed with decreased V-wave amplitude in the diabetic group. From the data above, the authors concluded that the function of the vestibulocochlear nerve is adequate and that the abnormalities are due to damage to more central structures [11]. Later, in a study where a similar age group was investigated, a higher incidence of hearing loss measured by pure tone audiometry (PTA), the amplitude of I-III-V waves was smaller in the course of ABR. The absolute latency of the I-II-III-V waves and the interpeak latency of the I-III, I-V waves were also delayed. The measurement found a positive correlation between the level of autonomic neuropathy examined and the brainstem function [12].

**Fig 1:**
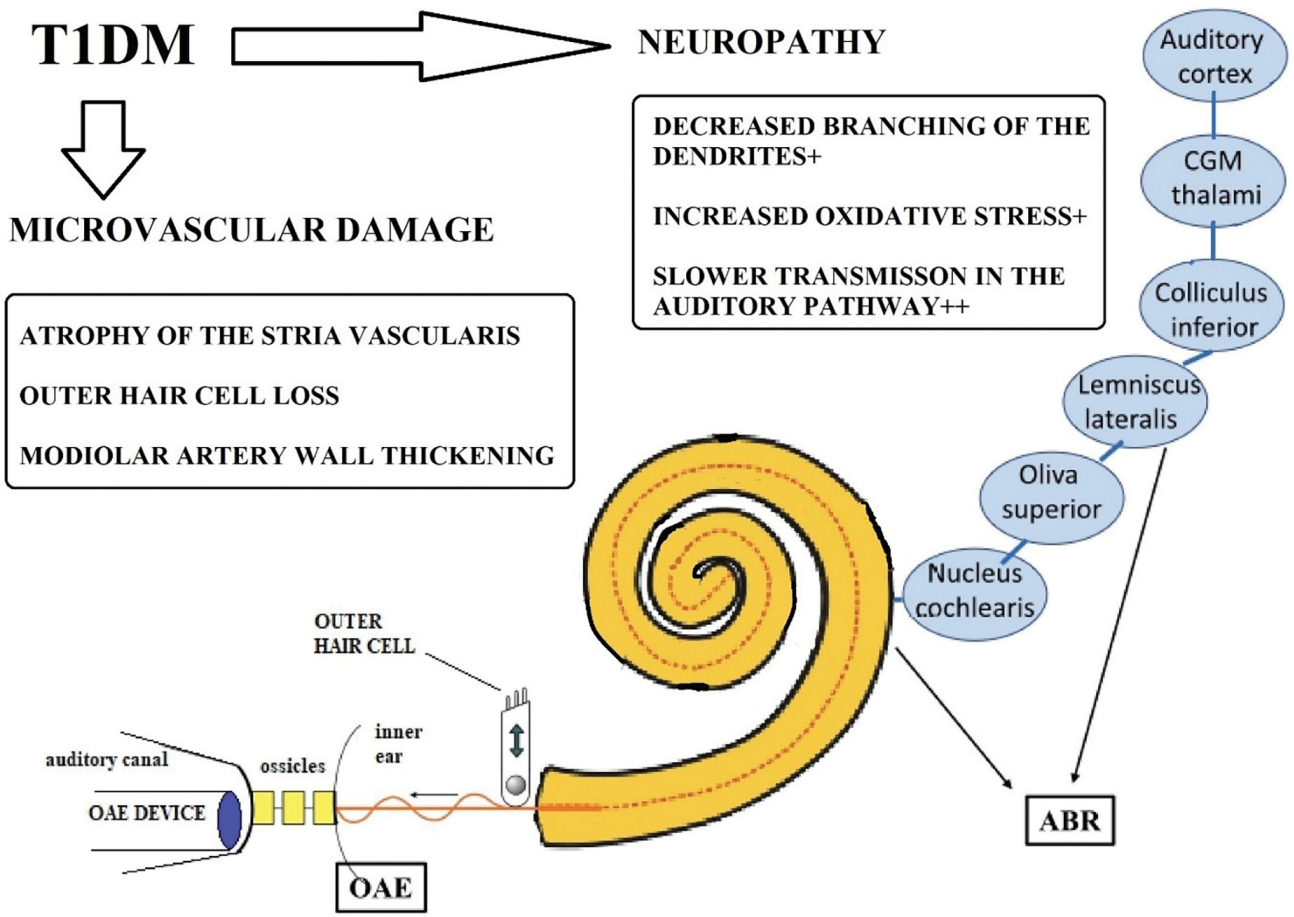
Potential development of hearing loss in type 1 diabetes mellitus (T1DM). Based on the results of previous studies [8, 9, 19–23], in T1DM, vasculopathy and neuropathy–both being common complications of T1DM–could have significant role in the development of hearing loss.

In addition to the above, microscopic histopathological examination of the temporal bone revealed differences in the samples from T1DM patients compared to non-diabetes individuals. The walls of vessels of the basilar membrane and of the stria vascularis were found to be thicker. In addition, the atrophy of the stria vascularis was also observed. The number of outer hair cells in the basal turn of the cochlea was significantly less. Loss of the spiral ligament cells was observed in the area of the middle and apical turn of the cochlea [8]. The cochlear spiral modiolar artery plays a crucial role in the arterial supply of the spiral ganglion. In T1DM, the thickening of the vessel wall and a higher vessel wall ratio compared to the vessel diameter have been observed [9]. Based on the above, it was concluded that in T1DM, the microvascular complications play a significant role in the mechanism of cochlea damage (Fig 1).

In a previous study, the evaluation of the PTA, ABR, and OAE results of young adults and T1DM patients (age between 17–46 years) showed the following alterations: Although the T1DM patients had significantly higher thresholds at 250, 1000, 2000, 4000, and 8000 Hz on the right and 250, 500, 1000, 4000, and 8000 Hz on the left ear than controls, the average PTA thresholds were less than 25 dB in both groups. Neural conduction in the auditory pathway was slowed down. Waves III and V were delayed and interpeak latencies between I-V waves were prolonged at both ears. 60% of the diabetics showed alterations by analyzing the otoacoustic emissions [20]. In the case of long-standing T1DM, the effect of the disease can be demonstrated even if an associated presbycusis is present. In the study of patients with T1DM and presbycusis over 60 years of age and subjects of the same age with exclusively age-related hearing loss, both absolute and interpeak latencies of auditory brainstem responses were found to be delayed in the T1DM study group. The amplitudes of the waves were also smaller than in the case of controls. No gender differences were found [13].

Using subjective and objective audiological methods, we tried to answer the question of whether T1DM and its complications accelerate the aging of the auditory system and to what extent are they causing hearing loss. Is the involvement of the inner ear or the damage to the central structures decisive in this population? The present study was designed to test these hypotheses that T1DM is one of the factors causing hearing loss, and to describe the characteristics of this hearing impairment.

## Materials and methods

### Patients and study design

The study was approved by the Regional and Institutional Committee of Science and Research Ethics, Semmelweis University (TUKEB 137-1/2010) and conducted in accordance with the regulations of the WMA Declaration of Helsinki and the General Data Protection Regulation issued by the European Union. All participants gave written informed consent prior any study specific procedures.

A prospective observational study was conducted at the Department. of Otorhinolaryngology, Head- and Neck Surgery, Semmelweis University, Budapest, Hungary, between 2017–2019. A total of 42 T1DM patients and 25 control subjects were included. T1DM patients were maintained and regularly checked-up at the Department of Internal Medicine and Hematology, Semmelweis University, Budapest, Hungary. The exclusion criteria included age < 18 years, occupational noise exposure, any sign of inflammation of the middle ear or even with acute upper airway infection the patients, and any previous ear diseases or other known disease related to the hearing system.

### Clinical data collection

Medical history data including co-morbidities and recent medications were collected. T1DM was confirmed using c-peptide, islet-cell- and/or glutamic acid decarboxylase antibody measurements. In the T1DM group, glycated hemoglobin (HbA_1C_) and creatinine level of patients was measured at the Central Laboratory of Semmelweis University. The estimated glomerular filtration rate (eGFR) was calculated using the Chronic Kidney Disease-Epidemiology Collaboration equations [24]. The duration of T1DM and common co-morbidities and T1DM-related complications, such as neuropathy, retinopathy, nephropathy and hyperlipidemia were recorded.

### Audiological investigations

All the patients went through a complete otorhinolaryngological investigation, including immittance audiometry, PTA, OAE and ABR measurements. No participants had history of occupational noise exposure. All procedures were followed in accordance with institutional guidelines, and every measurement were performed by the same study staff preventing collection bias.

#### Audiometry and middle ear evaluation

The initial hearing examination included tympanometry and PTA. Tympanometry was performed to check the status of the middle ear using a GSI Tympstar V2 tympanometer (Grason-Stadler Inc., Eden Prairie, MN, USA). We insert earplugs into the ear canals, which hermetically close them. Three tube goes through each earplug. They contain a tone loudspeaker, a microphone, and a pressure pump/manometer device. The acoustic admittance of the middle ear was measured by changing the air pressure in the ear canal and testing how the test tone (226Hz) is transmitted through the tympanic membrane and the ossicular chain. In general, healthy adults have the best tone transmission when the pressure in the ear canal is equal to the atmospheric pressure. In this case, the peak of the tympanogram is about 0 daPa If the pressure in the ear canal changes, the tone transmission gets less efficient [25]. All the investigated patients had normal middle ear pressure. One patient with Eustachian tube dysfunction has been excluded.

PTA measurements were evaluated in an audiological, sound-isolated cabin with a GSI 61 clinical audiometer (Grason-Stadler Inc., Eden Prairie, MN, USA). The hearing thresholds were measured with air conduction (AC) at 125 Hz, 250 Hz, 500 Hz, 1000 Hz, 2000 Hz, 4000 Hz, 8000 Hz and with bone conduction (BC) at 250 Hz, 500 Hz, 1000 Hz, 2000 Hz, 4000 Hz. Hearing impairment was detected when the threshold in either studied frequency was 25 dB or greater. The measurement procedure was performed according to the British Society of Audiology Guidelines.

#### Distortion product otoacoustic emission measurement

We used a Madsen Capella (Natus Medical Incorporated, Pleasanton, CA, USA) diagnostic OAE system to test distortion product OAE between 1000 and 8000 Hz: 1–1.5-2-3-4-6-8 kHz respectively. Otoacoustic emission is described by David Kemp [26]. This method is crucial for testing the function of the outer hair cells. These cells are responsible for the proper frequency discrimination and the amplification of sounds that are close to the hearing threshold.

**Fig 2:**
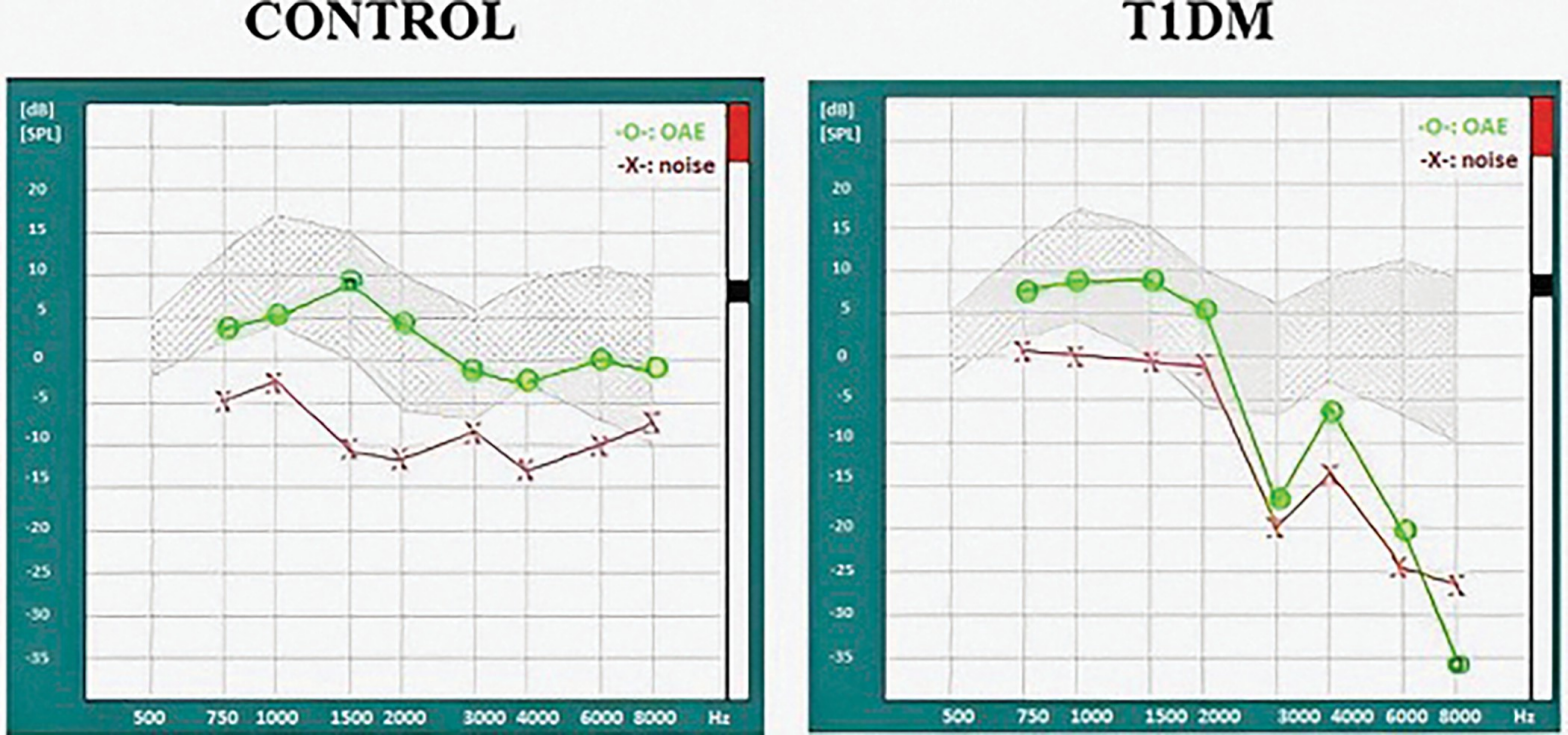
Distortion product otoacoustic emission (OAE) of a control subject and a T1DM patient from the same age group. The red line shows the background noise, while the green line shows the OAE values (dB SPL) at frequencies between 750 and 8000 Hz. The OAE in the control case is evocable at all measured frequencies, as the OAE values are in the grey/striped (normal) region. In contrast, in the T1DM patient has normal OAE response at the frequencies of 750–2000 Hz, but at 3000, 4000, 6000, 8000 Hz the OAE could not be evoked.

Distortion product otoacoustic emissions are based on that, two simultaneous voices (f1 and f2) are given into the external auditory canal. Healthy outer hair cells produce a specific tone that can be detected with a probe microphone beside the first two tones (f1 and f2). A higher tone is marked with f1. The exact frequency of the DPOAE can be calculated according to the following formula: 2f1-f2 [25]. The f2:f1 ratio was 1.22. The amplitudes of the primary tones were f1: 65 dB SPL and f2: 55 dB SPL respectively. When on a specific frequency the cochlea is damaged, we couldn’t detect proper emission. That’s an important differential diagnostic data. If the signal was above the limit defined for each frequency and the noise was below it, we registered an evocable OAE response at the specific frequency. The limits of each frequency were 4,5 dB sound pressure level (SPL) for 1000 Hz, 0 dB SPL for 1500Hz, -6 dB SPL for 2000 Hz, -7 dB SPL for 3000 Hz, -3 dB SPL for 4000 Hz, -7 dB SPL for 6000Hz, -10 dB SPL for 8000Hz (Fig 2).

#### Acoustically evoked brainstem response (ABR) measurement

**Fig 3:**
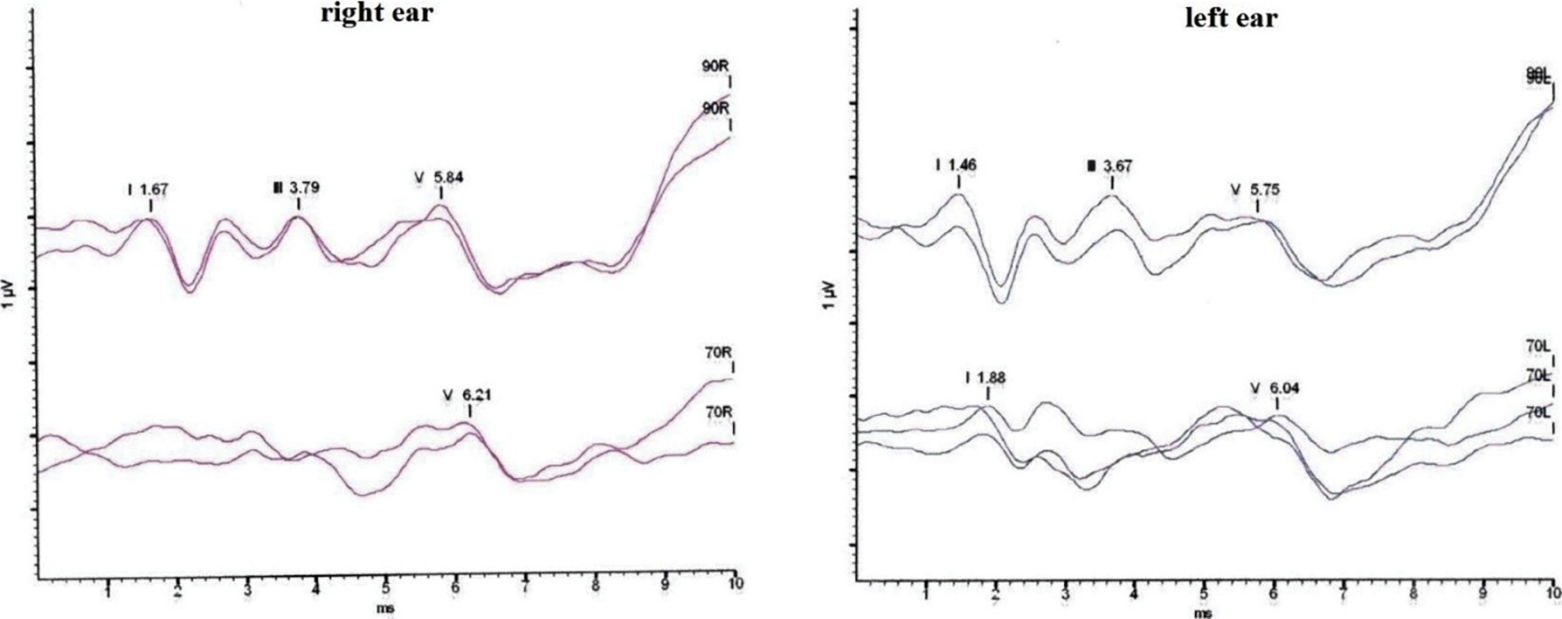
Acoustically evoked brainstem response waves of a type 1 diabetes patient. The upper two curves show the results obtained with 90 dB stimulation, whereas the curves on the bottom were generated from 70 dB stimulation. If the wave is present, its number (e.g. I, III, or V) and its latency is marked. I-III-V waves can be seen in both sides by 90 dB stimulus. By 70 dB stimulus, on the right side it is only the V wave and the I and V waves persist on the left side.

A GSI Audera (Grason-Stadler Inc., Eden Prairie, MN, USA) device was used to test ABR. We measured the brainstem responses with electrodes on the forehead and on the mastoids. The evoked responses were detected by giving 70 and 90 dB click tone into the ear canal through ear plugs. The measurements were performed in a soundproof room by using 2024 clicks of alternating polarity. The repetition rate of the stimuli was 33.1 Hz. The wave reproducibility was measured as well, which helped establish the reliability of the measurement. (See on Fig 3 the two lines for each stimulus intensities.) The impedance of the electrodes was in every case below 5 kΩ. The latencies of waves I, III, and V were measured and interpeak latencies were calculated. We considered a retrocochlear lesion if the absolute latencies of waves III and V or I-III and/or I-V interpeak latencies were prolonged and/or interaural latency differences were present and/or some of the III or V waves were absent and/or the form of the wave weren’t replicable. ABR latencies of the T1DM groups were compared to normative data (Fig 3).

### Statistical analysis

Statistical analysis was performed with R version 4.0.3 (R Foundation for Statistical Computing, Vienna, Austria, 2020). Welch’s two sample t-test, Fisher’s exact test and the Cochran–Mantel–Haenszel chi-squared test were used to compare groups. To determine the trends in pure tone threshold and otoacoustic emission, linear and logistic mixed effect models were used (R-packages nlme [27] and lme4 [28] respectively. P <0.05 was considered statistically significant and p-values were corrected with the Holm method [29] for multiple comparisons problem. Results were expressed as mean ± standard deviation and as the number of observations (percentage) for continuous and count data, respectively.

## Results

A total of 67 patients were included in the study, of whom 42 and 25 were T1DM patients and control subjects, respectively. The average age at the time of our investigation was 38.05 ± 10.73 in the T1DM group and 38.08 ± 9.53 in the Control group. Based on their age, the cases were subdivided into the following groups. Study subjects between the age of 19–39 years and 40–60 years were included in the T1DM_19-39_, T1DM_40-60_, Control_19-39_ and Control_40-60_ groups, respectively. Average age of study groups and number of included cases are summarized in Table 2.

**Table 2:** Patients’ data.

	T1DM patient (number)	T1DM age (year)	control cases (number)	control age (year)
age 19–39 years	26	31.19±5.42	12	29.67±6.08
age 40–60 years	16	49.19±7.05	13	45.85±3.44
summary, average	42	38.05±10.73	25	38.08±9.53

T1DM: type 1 diabetes mellitus.

The duration of T1DM was not statistically different between the two diabetes groups (p = 0.3309). Similarly, HbA_1C_ (p = 0.3148), creatinine (p = 0.6784) and eGFR (p = 0.2601) levels did not differ between the two patient groups. Of the investigated complications neuropathy was more pronounced in the T1DM_40-60_ group, compared to that of the T1DM_19-39_ group (p = 0.0015), while no difference in nephropathy (p = 0.6879), retinopathy (p = 1.0000) and hyperlipidemia (p = 0.4649) could be justified (Table 3).

**Table 3:** Clinical data of type 1 diabetes (T1DM) patients within the age groups of 19–39 and 40–60 years of age.

Clinical parameter	T1DM_19-39_ (n = 26)	T1DM_40-60_ (n = 16)	p-value
Age (years)	32.19 ± 5.54	49.19 ± 7.05	< 0.0001
Duration of diabetes (years)	14.73 ± 8.91	17.88 ± 10.61	0.3309
Type of T1DM			0.0161
• Conventional T1DM	24	9	
• LADA	2	7	
HbA_1C_ (%)	7.38 ± 1.50	7.90 ± 1.66	0.3148
Creatinine (μmol/L)	85.85 ± 35.21	82.00 ± 20.25	0.6784
Estimated glomerular filtration rate (mL/min/1.73 m^2^)	95.16 ± 24.63	85.35 ± 28.18	0.2601
Neuropathy	0 (0%)	6 (37.5%)	0.0015
Retinopathy	6 (23.1%)	2 (12.5%)	0.6879
Nephropathy	2 (7.7%)	1 (6.3%)	1.0000
Hyperlipidemia	5 (19.2%)	5 (31.3%)	0.4649

HbA_1C_: glycated hemoglobin; LADA: latent autoimmune diabetes in adults.

### Pure-tone audiometry

**Fig 4:**
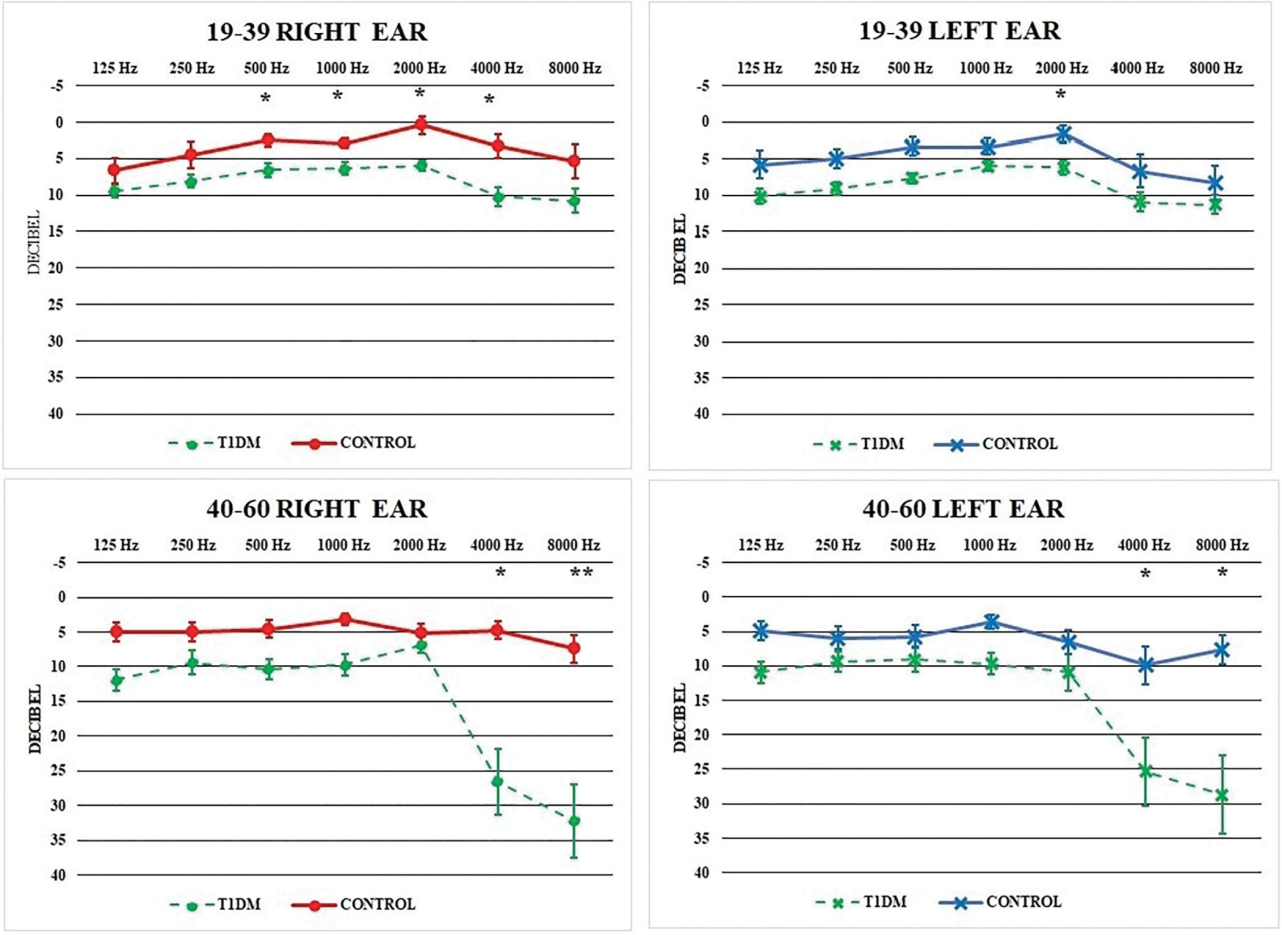
Mean hearing thresholds of the study groups. Top: 19–39 years-old groups, bottom: 40–60 years-old groups. T1DM: type 1 diabetes mellitus patients.

The incidence of hearing impairment was not different in the T1DM_19-39_ and Control_19-39_ groups, 15,4% and 16,6%, respectively. In contrast, hearing impairment was more common in the T1DM_40-60_ group (75%) than in the Control_40-60_ group (15,4%). In the diabetes study group the mean threshold values were higher in both age groups at all frequencies. In the T1DM_19-39_ group the hearing threshold in the middle to high frequency area (500–4000 Hz) on the right and in 2000 Hz on the left side were significantly higher than that of the Control_19-39_ group. The mean hearing thresholds in both groups were at all frequencies below 25 dB. Among 40–60 years-old study subjects, at 4000 and 8000 Hz, there was a significant difference between the T1DM_40-60_ and the Control_40-60_ results. In this high frequency region, the control subjects had a normal hearing, while the T1DM patients had usually a slight or mild hearing loss (n = 7), while some patients have had moderate or moderately severe (n = 3) or even severe (n = 2) hearing loss (Fig 4).

Analyzing the pure tone threshold altogether in a single statistical model showed that both T1DM (right ear: p < 0.0001, left ear: p = 0.0016) and older age (right ear: p < 0.0001, left ear: p = 0.0006) worsened the hearing of study subjects. According to the results of the model, the impaired hearing of the T1DM_19-39_ group is basically identical to that of the Control_40-60_ group, and the most impaired hearing was observed in the T1DM_40-60_ study group. No difference between left and right ear could be justified (p = 0.4275). Further analyses revealed that neither the duration of T1DM (p = 0.2950), nor actual HbA_1C_ levels (p = 0.3079) were associated with hearing loss, however, eGFR was significantly associated with hearing loss (p = 0.0285).

### Distortion product otoacoustic emissions

**Fig 5:**
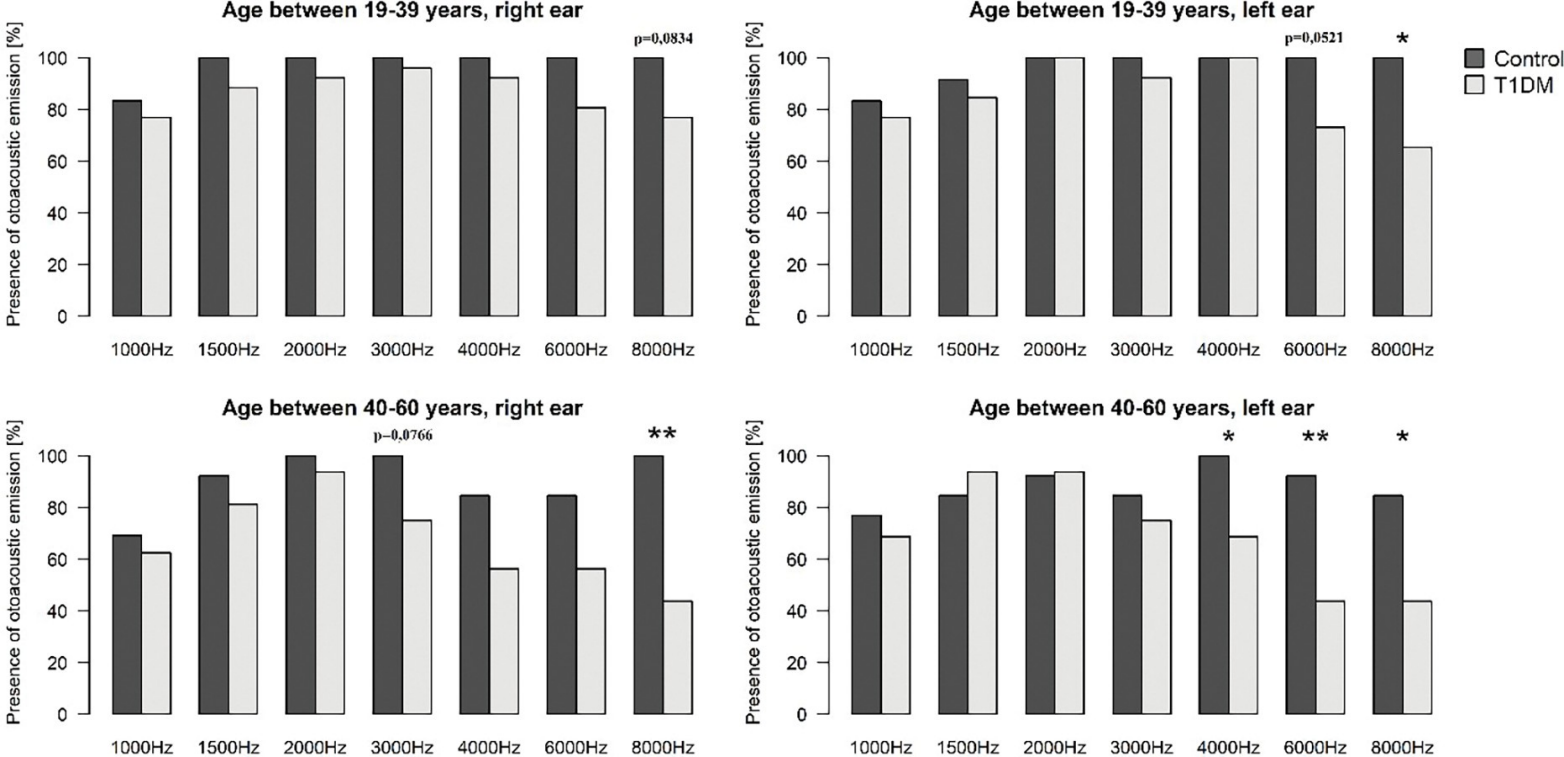
Otoacoustic emissions in the T1DM and control groups. At the higher frequencies there is a significant difference between the diabetes and the control groups. *: p< 0.05, **: p< 0.01.

To assess the loss of OAE, we had hypothesized greater effect in T1DM, therefore, one-sided tests were performed. Outer hair cell function of the Control_19-39_ and the T1DM_19-39_ groups was good at almost all frequencies, and only at 8000 Hz was a slight difference between the two groups (p < 0.05), where a moderate impairment in the left ear of the T1DM_19-39_ group was found. Diabetes patients of the 19–39 age group had tendentiously less OAE-s at 8000 Hz on the right (p = 0.0834) and at 6000 Hz on the left side (p = 0,.521). In contrast, OAE was severely impaired at the higher frequencies in the T1DM_40-60_ group, compared to that of the Control_40-60_ group: At 8000 Hz on the right side (p < 0.01) and at 4000 Hz (p <0.05), 6000 Hz (p < 0.01) and 8000 Hz (p < 0.05) on the left side. At 3000 Hz on the right side in the T1DM group, tendentiously fewer OAE responses could be detected (p = 0.0766; Fig 5).

The aggravating effect of T1DM (right ear: odds ratio (OR) = 6.46, p = 0.0006; left ear: OR = 4.90, p = 0.0013) on otoacoustic emission is more severe in both age groups than that of older age (right ear: OR = 4.14, p = 0.0028; left ear: OR = 2.87, p = 0.0125). Similarly to that of pure tone audiometry, sidedness (p = 0.7565), the duration of T1DM (p = 0.5438) and the actual HbA_1C_ levels (p = 0.6963) were not associated with OAE.

The mean amplitudes signal (dB SPL) and noise (dB SPL) level results of the OAE measurements were also compared between the study groups. No difference was found between the left and right ears (Signal: p = 0.5080; Noise: p = 0.6063). For both parameters, the clinically best results were found in the Control_19-39_ group, followed by the Control_40-60_ (Signal: –3.53 dB SPL, p = 0.1022; Noise: –2.62 dB SPL, p < 0.0001) and the two T1DM groups (Signal: –6.69 dB SPL, p < 0.0001; Noise: –2.10 dB SPL, p = 0.0004). The signal (–4.15 dB SPL, p = 0.0282) and noise (–2.46 dB SPL, p = 0.0030) level was significantly lower in the T1DM_40-60_ study group, compared to that of the T1DM_19-39_ group. The duration of T1DM (Signal: p = 0.7842; Noise: p = 0.5896) and the actual HbA1C levels (Signal: p = 0.4539; Noise: p = 0.2981) had no effect neither on the signal nor on the noise levels (Table 4).

**Table 4:** OAE signal and noise level results of the distortion product otoacoustic emission measurements in dB SPL.

	T1DM_19-39_ (n = 26)		T1DM_40-60_ (n = 16)	
Side of the ear	Right	Left	Right	Left
Signal (dB SPL)at				
• 1000 Hz	5.36 ± 4.71	6.07 ± 7.32	2.76 ± 7.39	10.03
• 1500 Hz	7.61 ± 5.83	6.13 ± 7.07	4.88 ± 8.85	7.06 ± 6.35
• 2000 Hz	3.97 ± 5.61	3.15 ± 7.00	3.63 ± 6.78	4.88 ± 7.10
• 3000 Hz	3.17 ± 5.14	1.68 ± 6.60	0.49 ± 9.86	1.14 ± 11.24
• 4000 Hz	7.46 ± 7.10	7.74 ± 5.19	–1.53 ± 12.40	–1.04 ± 12.48
• 6000 Hz	4.63 ± 10.62	1.33 ± 12.84	–4.01 ± 13.17	–5.59 ± 14.77
• 8000 Hz	1.53 ± 14.25	–0.12 ± 13.18	–5.93 ± 15.30	–7.37 ± 20.62
Noise (dB SPL) at				
• 1000 Hz	–6.21 ± 3.93	–5.14 ± 4.55	–8.39 ± 4.79	–7.08 ± 4.87
• 1500 Hz	–7.17 ± 4.70	–6.28 ± 4.38	–7.93 ± 5.26	–7.54 ± 4.87
• 2000 Hz	–8.78 ± 4.12	–8.92 ± 3.97	–9.71 ± 4.96	–9.61 ± 4.93
• 3000 Hz	–9.89 ± 3.63	–9.33 ± 8.18	–13.28 ± 5.61	–12.22 ± 6.14
• 4000 Hz	–9.63 ± 4.17	–8.29 ± 6.09	–11.78 ± 9.01	–13.45 ± 5.12
• 6000 Hz	–9.51 ± 5.58	–11.74 ± 6.54	–12.38 ± 5.70	–13.82 ± 6.69
• 8000 Hz	–9.25 ± 9.58	–10.85 ± 7.91	–13.32 ± 9.15	–14.86 ± 10.72
	Control_19-39_ (n = 12)		Control_40-60_ (n = 13)	
Side of the ear	Right	Left	Right	Left
Signal (dB SPL)at				
• 1000 Hz	7.12 ± 6.33	8.21 ± 3.84	5.62 ± 6.25	5.47 ± 5.24
• 1500 Hz	10.62 ± 6.00	7.94 ± 6.45	8.15 ± 5.43	8.19 ± 6.49
• 2000 Hz	6.20 ± 5.58	7.33 ± 6.13	6.88 ± 6.09	4.77 ± 8.18
• 3000 Hz	8.68 ± 4.84	8.03 ± 4.79	5.68 ± 6.39	4.65 ± 8.00
• 4000 Hz	11.98 ± 2.71	10.73 ± 4.63	8.02 ± 7.12	9.26 ± 6.25
• 6000 Hz	15.45 ± 6.96	10.05 ± 8.42	6.57 ± 10.04	10.67 ± 9.56
• 8000 Hz	15.45 ± 8.55	16.55 ± 11.50	7.42 ± 9.58	8.52 ± 12.91
Noise (dB SPL) at				
• 1000 Hz	–4.08 ± 7.04	–5.63 ± 4.75	–7.70 ± 3.84	–8.58 ± 2.86
• 1500 Hz	–7.55 ± 3.06	–7.24 ± 4.28	–10.29 ± 2.78	–9.31 ± 4.39
• 2000 Hz	–7.82 ± 8.10	–6.84 ± 7.65	–9.62 ± 4.21	–11.59 ± 4.15
• 3000 Hz	–8.59 ± 5.55	–9.10 ± 3.37	–11.88 ± 3.10	–11.82 ± 4.13
• 4000 Hz	–9.42 ± 7.35	–10.34 ± 1.48	–11.44 ± 3.31	–12.40 ± 3.89
• 6000 Hz	–5.03 ± 5.54	–3.80 ± 5.35	–7.45 ± 4.74	–8.27 ± 3.31
• 8000 Hz	–1.20 ± 2.97	–2.15 ± 4.58	–4.58 ± 4.29	–4.22 ± 4.92

### Acoustically evoked brainstem response

ABR waves were analyzed only in the diabetes patient groups. A possible retrocochlear lesion arose in 15% and 25% of the T1DM_19-39_ and T1DM_40-60_ patients (p = 0.4538), respectively.

Latency data of waves 1, 3 and 5, and interpeak latencies of waves 1–3 and 3–5 at 70 dB and 90 dB were compared. It was found that wave 1 at 70 dB in average had a ~0.06 milliseconds longer latency in the right ear of the patients (p = 0.0213). No further differences could be found regarding the 70 dB measurements. In the 90 dB measurements wave 1 was affected by sidedness (latency of the right ear: + 0.042 milliseconds; p = 0.0358) and age (latency in the T1DM_40-60_ group: + 0.089 milliseconds; p = 0.0167). Wave 3 was significantly and marginally affected by higher HbA_1C_ values (every 1% HbA_1C_ increase: + 0.028 milliseconds; p = 0.0119) and age (latency in the T1DM_40-60_ group: + 0.065 milliseconds; p = 0.0669), but no difference was found regarding sidedness. And in wave 5 only sidedness affected latencies (latency of the left ear: + 0.067 milliseconds; p = 0.0032). No additional effector could be identified when investigating interpeak latencies. Averaged latency data of the four study groups are summarized in Table 5.

**Table 5:** Acoustically evoked brainstem latencies (in millisecond) of type 1 diabetes patients in the two age groups.

	T1DM_19-39_ (n = 26)		T1DM_40-60_ (n = 16)	
Side of the ear	Right	Left	Right	Left
70 dB				
• Wave I	1.80 ± 0.11	1.75 ± 0.15	1.77 ± 0.15	1.67 ± 0.14
• Wave III	3.84 ± 0.15	3.82 ± 0.13	4.03 ± 0.75	3.87 ± 0.20
• Wave V	5.78 ± 0.23	5.77 ± 0.20	5.86 ± 0.28	5.83 ± 0.22
• Interpeak I-III	2.03 ± 0.15	2.08 ± 0.12	2.26 ± 0.79	2.18 ± 0.24
• Interpeak III-V	1.91 ± 0.14	1.94 ± 0.15	1.77 ± 0.75	1.94 ± 0.19
90 dB				
• Wave I	1.45 ± 0.15	1.40 ± 0.09	1.52 ± 0.12	1.51 ± 0.10
• Wave III	3.55 ± 0.09	3.56 ± 0.09	3.62 ± 0.16	3.66 ± 0.15
• Wave V	5.43 ± 0.17	5.47 ± 0.16	5.53 ± 0.25	5.65 ± 0.38
• Interpeak I-III	2.10 ± 0.14	2.17 ± 0.12	2.09 ± 0.19	2.15 ± 0.15
• Interpeak III-V	1.88 ± 0.13	1.91 ± 0.16	1.87 ± 0.14	1.91 ± 0.20

## Discussion

The present study demonstrated a positive association between age, T1DM and hearing loss on three different modalities of hearing measurement. Our findings provide support for the concept that T1DM is one of the factors which can damage the hearing system, and this negative effect gets worse with aging. We implemented complex audiological investigations in a single study. To our knowledge, most previous studies focused one or two hearing testing methods only. E.g., in the meta-analysis of Teng et al. [30] a higher incidence of hearing loss in T1DM patients has been reported. When the frequencies were analyzed separately, there was only a significant difference at 4000 Hz. They have underlined that all PTA measurements averaged below 25 dB and ABR waves III and V have been delayed and interpeak latencies I-III and I-V have been lengthened. It has to be noted, however, that compared to our study, Teng et al. did not analyze OAEs as an important marker of the proper inner ear function [30]. In a similar meta-analysis, Mujica-Mota et al. have reported a 7.7x greater risk to have hearing loss if the patient had T1DM and significantly lower OAE amplitudes and longer I, III and V ABR wave latencies have been found among T1DM patients. They have claimed that diabetes is a risk factor for hearing loss, and the disease may influence negatively the auditory organ more over time. The authors underlined a similar hypothesis to our work that the cochlea and the neurotransmission of the vestibulocochlear nerve are affected by diabetes [19].

Hearing loss occurs more often in diabetes and a few hypotheses have emerged as to what might be behind this phenomenon including but not limited to role of glycated metabolites, microvascular causes and oxidative stress. The detrimental effect of glycated metabolites is theoretically based on the positive correlation between HbA_1C_ levels and the increase of the subjective hearing threshold measured at 250–500 Hz in the case of patients with diabetes. The correlation between blood sugar level and the hearing threshold changes (at 250 and 500 Hz) have also been showed [31]. A possible connection between microangiopathy and hearing loss was that the degree of hearing loss was positively correlated with the serum creatinine value–which might refer to nephropathy–in diabetes patients with sensorineural hearing impairment [32]. During a study on Akita mice which are specific models for insulin dependent diabetes mellitus, elevated hearing thresholds were found compared to wild-type mice. During the histological processing of their cochlea, a smaller number of ganglion spirale neurons, a thinner stria vascularis, and differences in capillaries were observed in the Akita mice [33]. These findings are similar to the previous analyses of the temporal bone of T1DM patients [8]. In their review, Helzner and Contrera have shown the role of neuropathy. They have hypothesized that high blood sugar levels cause nerve damage by reducing the branching of the dendrites through oxidative stress. The hypothesis is also supported by histopathologic lesions, animal experiments with rats and the increased latencies of brainstem evoked potentials [22]. Moreover, the role of reactive oxygen species has also emerged as a factor of hearing loss in diabetes. In a study of T2DM patients without microangiopathic complication(s), higher levels of antioxidant superoxide activity and oxidized protein products were found within the diabetes group. In the subgroup of T2DM patients with hearing impairment, the measured superoxide dismutase activity and oxidized protein product levels have been even higher than in those T2DM patients without hearing impairment. Furthermore, a negative correlation was found between the level of nitric oxide (NO), vitamins C and E, and the degree of hearing loss in the T2DM group [23]. Animal studies have shown that NO plays a role in the vasodilatation of cochlear vessels, and so it is essential for cochlear blood flow regulation [34, 35]. Diabetes gives rise to endothelial damage and lower NO levels. Among other factors, this causes increased oxidative stress and may contribute to cochlear injury [36]. It has to be noted, however, that the role of glycated metabolites, microvascular cause, and oxidative stress in T2DM-related hearing impairment is better researched than in T1DM. Further research may target the role of oxidative stress in type 1 diabetes both in animal models and humans. The possible correlation of nitric-oxide, oxidized protein products, antioxidant agents, and objective hearing measurements, for example, otoacoustic emissions is still to discover.

In our investigation, hearing loss was the most frequent in the T1DM_40-60_ group than in control subjects of the same age. In the non-T1DM group the mean hearing thresholds were in the normal hearing region of all studied frequencies. Although in the T1DM_19-39_ group we could detect significantly higher thresholds (right ear: 500Hz-4kHz, left ear: 2 kHz), the mean hearing thresholds were also in the normal ranges. A similar observation was reported by Hou et al. They found that the mean thresholds of diabetes patients in any studied frequencies were less than 25 dB [20]. The T1DM_40-60_ group had significantly worse hearing on 4 and 8 kHz, on both the right and left ear than the controls. This result differs from the results of Celik et al., who reported significantly higher hearing thresholds among T1DM patients at all measured frequencies [37]. Most of our T1DM_40-60_ patients usually had a slight or mild hearing loss on 4–8 kHz. It is important to note, that moderate and severe hearing loss also occurred. Other publications also reported that hearing loss occurs more at higher frequencies [21, 37]. All our statistical models supported the hypothesis that the presence of T1DM made the hearing of these patients worse. The T1DM_40-60_ group had a significantly poorer outer hair cell function at 8 kHz than the corresponding aged control group. A similar observation was made for the left ear of T1DM_19-39_ patients. The ABR analysis showed, that a notable proportion of diabetes patients had signs of a retrocochlear lesion. Prolonged absolute and interpeak latencies may be a sign of such damage. Prolonged wave V latency and I-V interpeak latency have been described in other reports [20, 21]. It has to be highlighted, however, that the duration of diabetes was only slightly longer in the T1DM_40-60_ group, but without statistical difference, suggesting that this observation might be a combined effect of aging and T1DM, but the role of the different T1DM subtypes (conventional T1DM vs. LADA) should be also considered as a significant proportion of the patients in the T1DM_40-60_ had LADA. An interesting observation of our study was that the right and left ear was differently affected in the T1DM groups, the most impairment was found in the right ear of the patients. We consider further investigations necessary to find out what could be behind this observation.

Interventions to prevent, identify and address hearing loss are cost-effective and can bring great benefit to individuals. These are the main purposes of newborn hearing screening programs around the world. From birth the aging of the hearing system depends on genetics and other intrinsic and extrinsic factors. The starting point can be detected by the newborn hearing screening [38]. People with hearing loss benefit from early identification; use of hearing aids, cochlear implants and other assistive devices; captioning and sign language; and other forms of educational and social support. In recent years, a considerable amount of scientific evidence has been published highlighting the connection between hearing loss and mental health. It’s clear that there is an association between unassisted hearing loss and cognitive decline and dementia. Why this should be the case is still not clear and much research is being undertaken to try and establish how and why hearing loss and cognitive health are connected. Important research findings published in The Lancet in 2017 stated that one in three cases of dementia could be prevented if people managed a number of lifestyle factors in midlife (between ages 40 to 65) including hearing loss [39]. Unmanaged hearing loss adds to the brain’s cognitive load and can lead to social isolation and depression, good reasons to care for your hearing and watch for signs that your hearing may be changing (in just the same way as you’d care for eyesight and dental health). Particularly after the age of 40, hearing tests should be as routine a part of healthcare as vision tests and dental checks.

### Limitations of the study

This study has some limitations, including the high heterogeneity of patients, and the small sample sizes. Number of control subjects was even smaller than that of the diseased group.

## Conclusions

Summarizing the result of the current study, changes of the hearing system caused by type 1 diabetes mellitus can be detected in the young adulthood and are more pronounced with aging. The cochlear and the retrocochlear parts of the auditory system are also affected.
